# Supplementary material for: Structural basis for catalysis by human lipoyl synthase
Source: Nat Commun. 2025 Jul 10;16:6355. doi: 10.1038/s41467-025-61393-x (PMC12246082; doi:10.1038/s41467-025-61393-x)
Supplement: Supplementary file 1 — Supplementary Information [file 41467_2025_61393_MOESM1_ESM.pdf]

Supplementary Information for:

Structural basis for catalysis by human lipoyl synthase

Authors: Olga A. Esakova[1,*], Douglas M. Warui[1], Syam Sundar Neti[1], John N. Alumasa[1,4] and Squire J. Booker[1,2,3*]

Affiliations: [1]Department of Chemistry, [2]Department of Biochemistry & Molecular Biology, and the [3]Howard Hughes Medical Institute, The Pennsylvania State University, University Park, Pennsylvania 16802, USA

[4]Present address: Department of Chemistry and Biochemistry, Miami University, Oxford, OH. 45056

Corresponding authors: squire@psu.edu, oae3@psu.edu

TABLE of CONTENTS

Page 2. Table of contents

Page 3. Supplementary Note 1 Abbreviations

Page 4. Supplementary Table 1

Page 5. Supplementary Table 2. Analysis of the protein-protein interactions between LIAS and H_{pro}

Page 6. Supplementary Fig. 1. Superimposed structures of LIAS with *Mt*LipA and *Te*LipA

Page 7. Supplementary Fig. 2. Structures of LIAS showing the presence of a loop comprising residues 118-126

Page 8. Supplementary Fig. 3. Structures of LIAS-6S-OCT-H_{pro} covalent complex

Page 9. Supplementary Fig. 4. The overall structure of human H_{pro}

Page 10. Supplementary Fig. 5. Protein-protein interactions between LIAS and H_{pro} in the LIAS-6S-OCT-H_{pro} covalent complex

Page 11. Supplementary Fig. 6. Analysis of conserved residues in the LIAS-6S-OCT-H_{pro} covalent complex

Page 12. Supplementary Fig. 7. Superimposed structures of the E2 subunits of the BCODC, KGC, and PDC

Page 13. Supplementary Fig. 8. The interaction of conserved residues of LIAS involved in NKH

Page 14. Supplementary References

Supplementary Note 1 ABBREVIATIONS

5'-dA•, 5'-deoxyadenosyl 5'-radical; 5'-dAH, 5'-deoxyadenosine; aa, amino acid; H•, hydrogen atom; ACP, acyl carrier protein; BCODC, branch chain oxo-acid dehydrogenase complex; BME, β-mercaptoethanol; DTT, dithiothreitol; FeS, iron-sulfur; GCS, glycine cleavage system; HEPES, *N*-(2-Hydroxyethyl)-piperazine-*N*'-(2-ethanesulfonic acid); H_{pro}, H protein; IMAC, immobilized metal affinity chromatography; IPTG, isopropyl β-D-thiogalactopyranoside; KDC, α-ketoglutarate dehydrogenase complex, KPB, potassium phosphate buffer; LA, lipoic acid; LCP, lipoyl carrier protein; LD, lipoyl domain; LIAS, human lipoyl synthase; LplA, lipoate protein ligase A; LipA, *E. coli* lipoyl synthase; LipB, octanoyl protein ligase B; LipCo, lipoyl cofactor; Lip-H_{pro}, lipoyl-H protein; LIPT1, human lipoyl transferase 1; LIPT2, human octanoyltransferase; Met, methionine; *Mt*LipA, *Mycobacterium tuberculosis* lipoyl synthase; NTA, Ni-nitrilotriacetic acid; OCT, octanoyl; OCT-8_{mer}, octanoylated 8_{mer} peptide substrate containing an octanoyllysyl moiety; OCT-ACP, octanoyl-acyl carrier protein; OCT-H_{pro}, octanoyl-H protein; PDC, pyruvate dehydrogenase complex; PLP, pyridoxal 5'-phosphate; PMSF, phenylmethylsulfonylfluoride; RS, radical SAM; SAM, *S*-adenosylmethionine; SAH, *S*-adenosylhomocysteine; SDS-PAGE, sodium dodecyl sulfate–polyacrylamide gel electrophoresis; *Te*LipA, *Thermosynechoccus elongatus* lipoyl synthase; TCEP•HCl, tris(2-carboxyethyl)phosphine hydrochloride; Trp, tryptophan.

Supplementary Table 1. Data collection and refinement statistics

	LIAS Fe-peak	LIAS Native	LIAS 5'dAH+Met OCT-8-mer	LIAS-H protein SAM	LIAS-H protein 5'dAH+Met
**Data collection**					
Wavelength	1.72	0.979	0.979	0.979	0.979
Space group		I 2 2 2	P 21	P 21 21 21	P 21 21 21
Cell dimensions					
a, *b*, *c* (Å)	69.05, 89.54, 108.39	68.35, 88.79, 108.17	48.54, 69.03, 93.79	68.69, 97.12, 80.60	48.68, 169.21, 183.18
α, β, γ (°)	90, 90, 90	90, 90, 90	90, 94.5, 90	90, 108.1, 90	90, 90, 90
Resolution (Å)	50-2.52 (2.56-2.52)*	50-1.54 (1.60-1.54)*	50-1.58 (1.61-1.58)*	50- 1.50 (1.53-1.50)*	50-2.45 (2.49-2.45)*
R_{sym} or R_{merge}	0.129 (0.363)	0.051 (0.613)	0.087 (0.985)	0.055 (0.224)	0.142 (0.989)
$I / \sigma I$	15.3 (6.14)	20.9 (2.0)	20.5 (1.2)	12.5 (2.6)	9.8 (1.2)
Completeness (%)	99.1 (100)	98.8 (98.6)	98.6 86.2)	89.3 (83.4)	98.0 (89.7)
Redundancy	9.6 (6.7)	4.1 (3.8)	7.0 (4.8)	2.0 (1.7)	5.9 (4.4)
**Refinement**					
Resolution (Å)		25.9-1.54	27.77-1.58	41.02-1.50	33.36-2.45
No. reflections		47046	71880	143280	50290
R_{work} / R_{free}		0.1635/0.1878	0.1652/0.2020	0.1388/0.1595	0.1858/0.2197
No. atoms					
Protein		2426	4688	7739	9948
Ligand/ion		60	59	131	216
Water		372	623	1474	196
B-factors					
Protein		19.3	24.9	16.5	49.3
Ligand/ion		20.0	19.6	12.2	43.6
Water		32.3	32.9	29.6	39.0
R.m.s. deviations					
Bond lengths (Å)		0.004	0.008	0.015	0.007
Bond angles (°)		0.82	0.88	1.41	0.65

*Values in parentheses are for the highest-resolution shell.

Supplementary Table 2. Analysis of the protein-protein interactions between LIAS and H-protein

LIAS		H-protein	
**Hydrophobic interactions**			
73	Arg	83	Val
76	Leu	109	Ala
84	Ile	74	Phe
84	Ile	75	Ala
84	Ile	78	Ala
84	Ile	79	Leu
85	Pro	78	Ala
86	Met	167	Tyr
86	Met	168	Ile
86	Met	74	Phe
90	Tyr	74	Phe
91	Asn	169	Lys
310	Met	107	Lys
311	Gln	106	Val
312	Pro	106	Val
312	Pro	107	Lys
313	Thr	106	Val
**Hydrogen bonds**			
75	Arg	104	Glu
76	Leu	104	Glu
87	Gly	170	Ser
88	Lys	170	Ser
91	Asn	168	Ile
91	Asn	170	Ser
110	Arg	77	Glu
314	Arg	81	Asp
**Ionic interactions**			
73	Arg	104	Glu
75	Arg	104	Glu
81	Lys	104	Glu
81	Lys	111	Glu
110	Arg	81	Asp
314	Arg	81	Asp
315	Arg	81	Asp
**Sulphur-aromatic interactions**			
86	Met	167	Tyr
**Cation-Pi interactions**			
75	Arg	84	Tyr
94	Lys	74	Phe

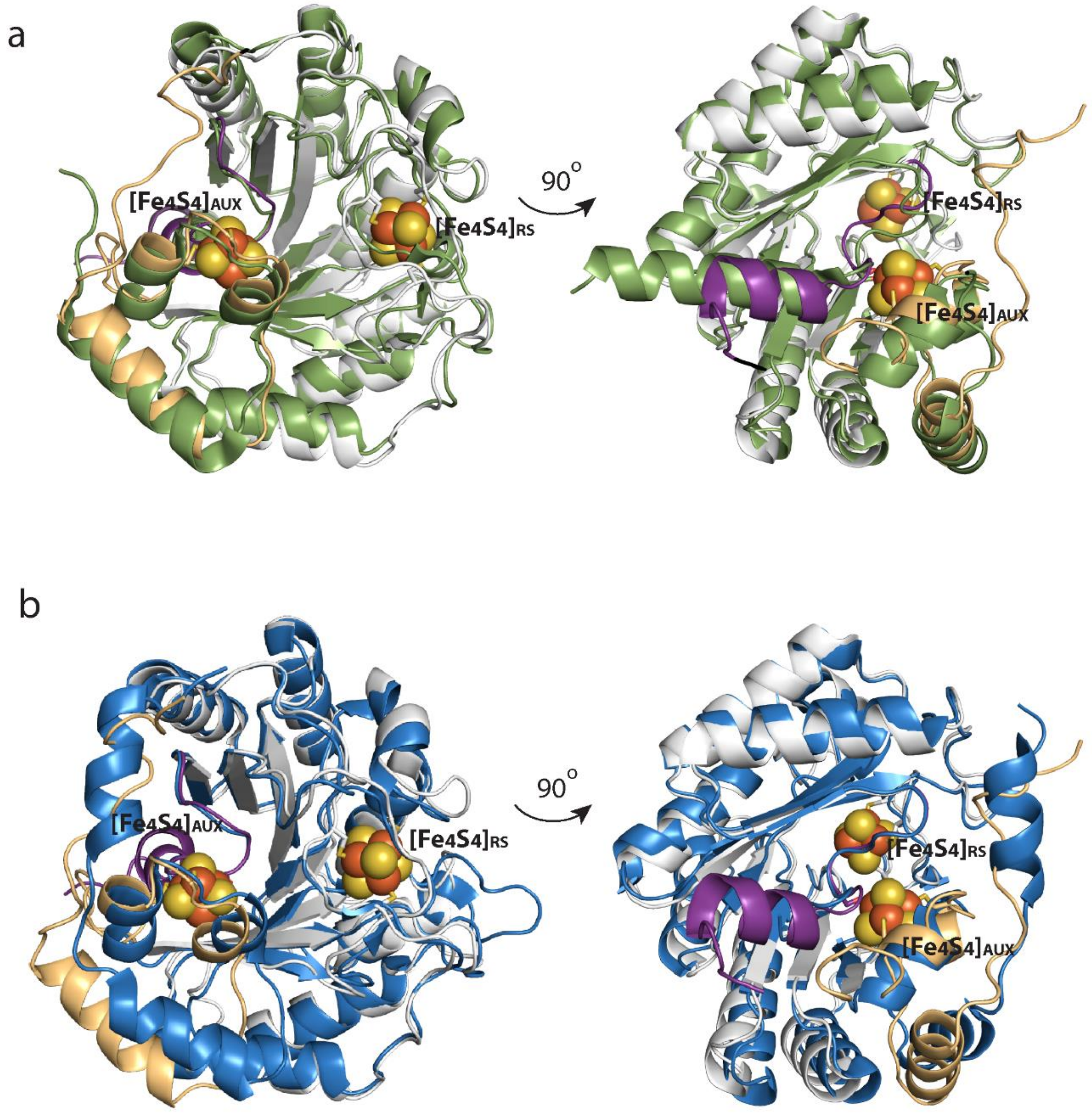

Supplementary Fig. 1. Superimposed structures of LIAS with *Mycobacterium tuberculosis* lipoyl synthase (*Mt*LipA) and *Thermosynechoccus elongatus* lipoyl synthase (*Te*LipA). Cartoon representation of the superimposed structures of (**a**) LIAS and *Mt*LipA (green PDB ID: 5EXJ); and (**b**) LIAS and *Te*LipA (blue PDB ID: 4U0P). For clarity, only the [Fe_4S_4] clusters in LIAS are shown. LIAS color code: N-terminal domain (tan); radical SAM (RS) domain (light grey); C-terminal domain (purple). Iron–sulfur atoms of the auxiliary ([Fe_4S_4]$_{AUX}$) and radical SAM ([Fe_4S_4]$_{RS}$) clusters are represented as orange and yellow balls, respectively.

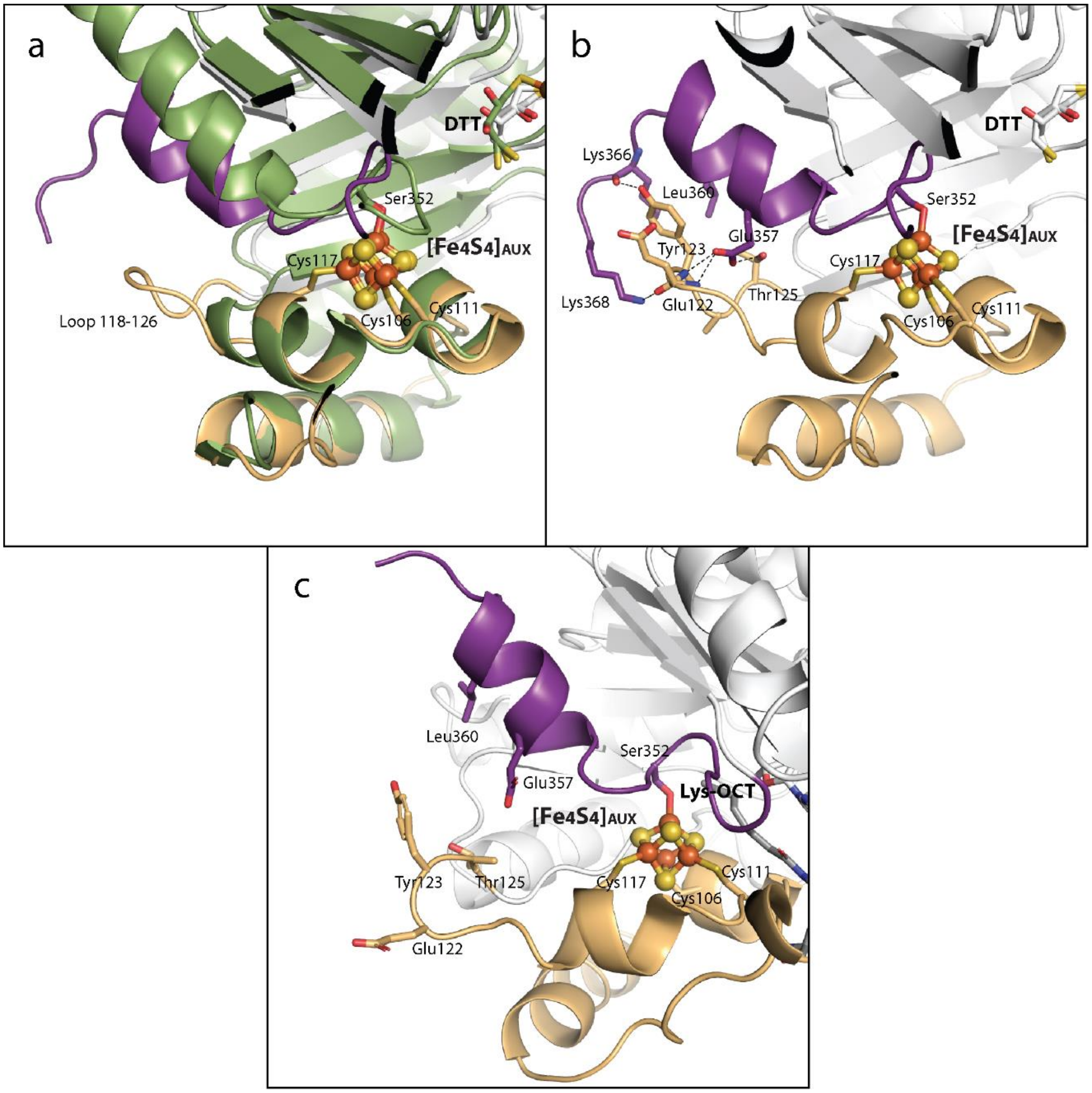

Supplementary Fig. 2. **Structures of LIAS showing the presence of a loop comprising residues 118-126.** Cartoon representation of (**a**) the superimposed LIAS and *Mycobacterium tuberculosis* lipoyl synthase (green PDB ID: 5EXJ) structures; (**b**) LIAS structure with dithiothreitol (DTT) bound to the cluster; (**c**) LIAS in the presence of the octanoylated 8_{mer} peptide substrate (Lys-OCT) and 5'-deoxyadenosine (5'-dAH) + Met (not shown). LIAS color code: N-terminal domain (tan); radical SAM (RS) domain (light grey); C-terminal domain (purple). Iron–sulfur atoms of the auxiliary ($[Fe_4S_4]_{AUX}$) cluster are represented as orange and yellow balls, respectively.

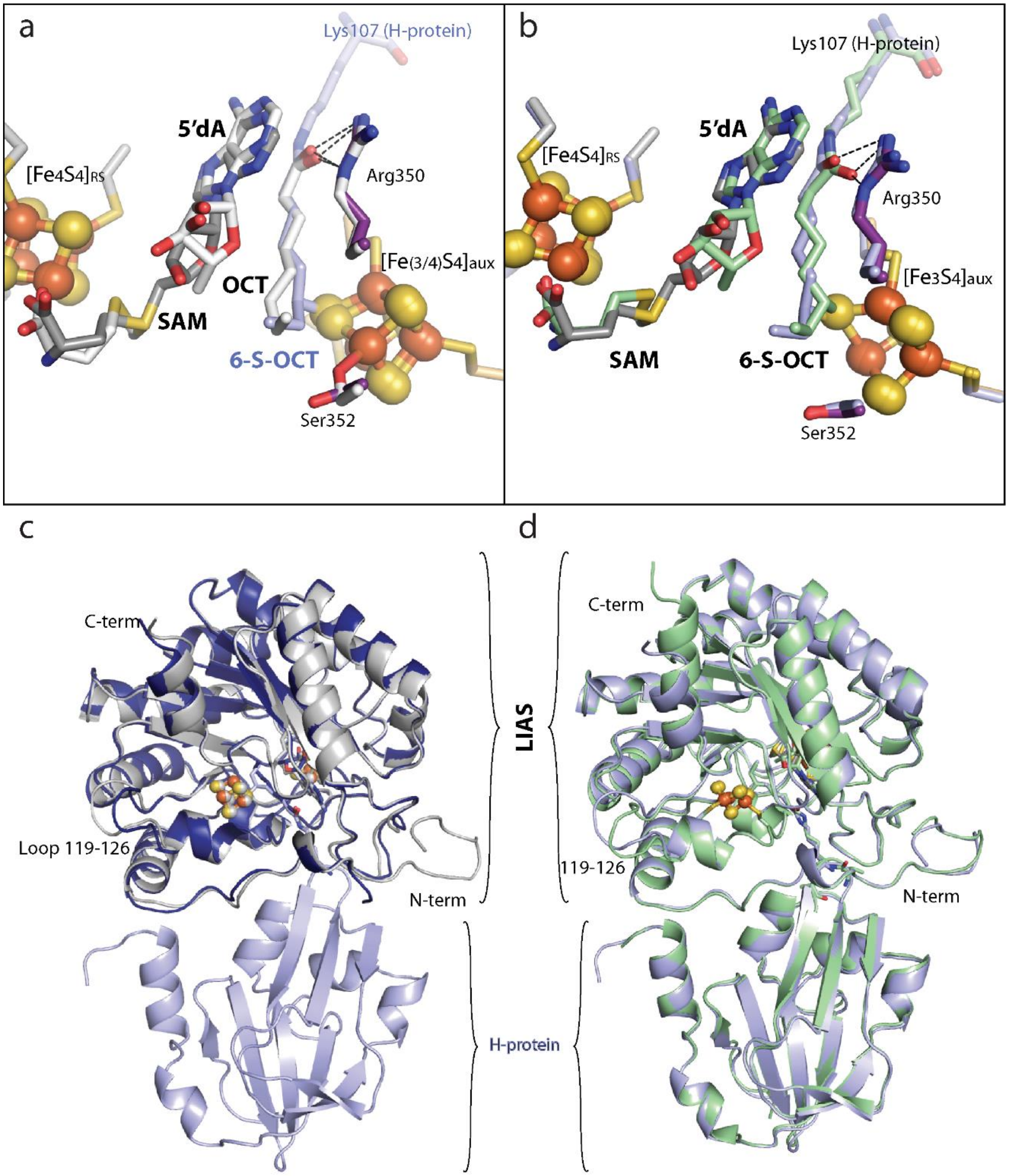

Supplementary Fig. 3. **Structures of the covalent complex of LIAS and 6-mercaptooctanoyllysyl H-protein (6S-OCT-H$_{pro}$)**. Cartoon representation of (**a**) the overlaid active sites of LIAS in the presence of the octanoylated 8$_{mer}$ peptide substrate (OCT-8$_{mer}$), and 5’-deoxyadenosine (5’-dAH)+Met (white) and the LIAS-6S-OCT-H$_{pro}$ covalent complex in the presence of S-adenosylmethionine (SAM) (light blue); (**b**) the overlaid active sites of the LIAS-6S-OCT-H$_{pro}$ complex in the presence of SAM (light blue) and in the presence of 5’-dAH+Met (H$_{pro}$ in light green). LIAS color code: N-terminal domain (tan); radical SAM (RS) domain (light grey); C-terminal domain (purple); iron–sulfur atoms of the auxiliary ([Fe_4S_4]$_{AUX}$) and radical SAM ([Fe_4S_4]$_{RS}$) clusters are represented as orange and yellow balls, respectively. Cartoon representation of superimposed structures of (**c**) LIAS in the presence of the OCT-8$_{mer}$ substrate and 5’-dAH+Met (dark blue) and the LIAS-6S-OCT-H$_{pro}$ complex in the presence of 5’-dAH+Met (LIAS in light grey and H$_{pro}$ in light green); (**d**) LIAS-6S-OCT-H$_{pro}$ complex in the presence of SAM (light blue) and in the presence of 5’-dAH+Met (light green). There is a displacement of the loop containing residues 119-126 in structures containing SAM.

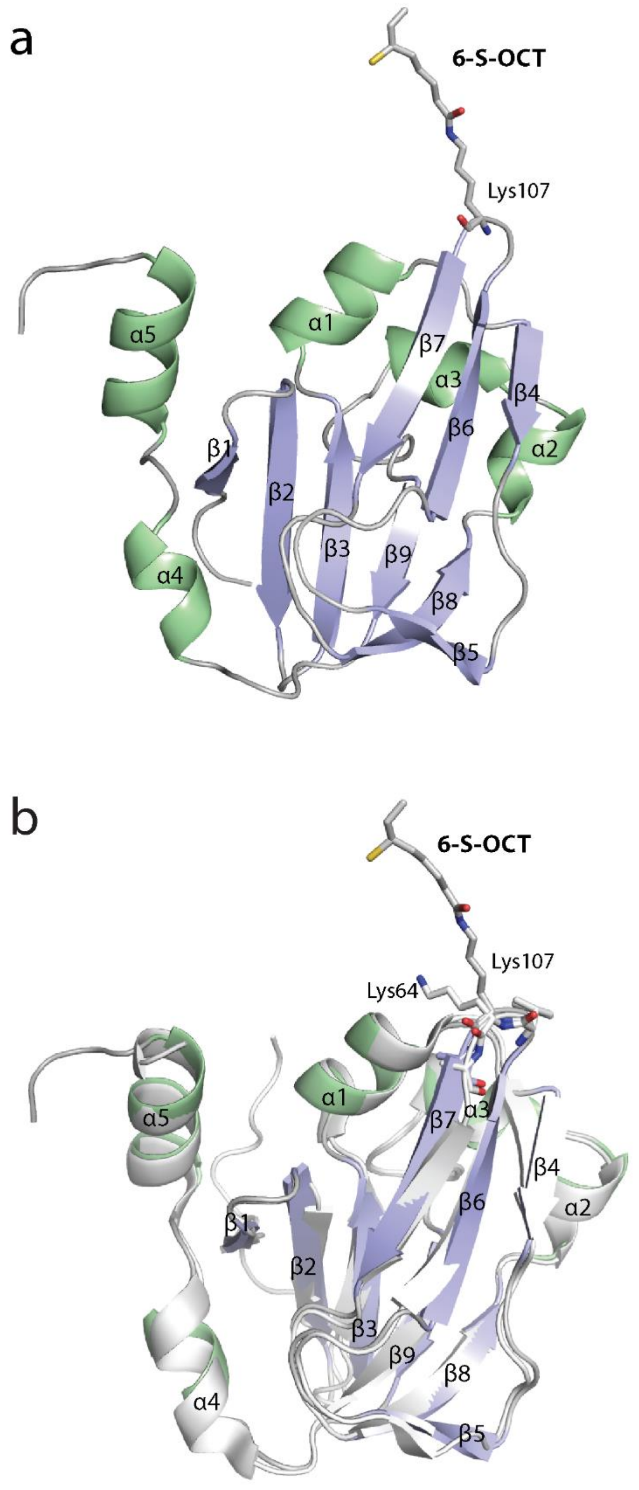

Supplementary Fig. 4. **The overall structure of human H protein (H_{pro})**. Cartoon representation of (**a**) the overall human 6-mercaptooctanoyllysyl (6-S-OCT)-H_{pro} structure (α-helices (green) and β-strands (light blue)); (**b**) the superimposed human H_{pro} and *E. coli* H_{pro} (white, PDB ID: 3A7L) structures.

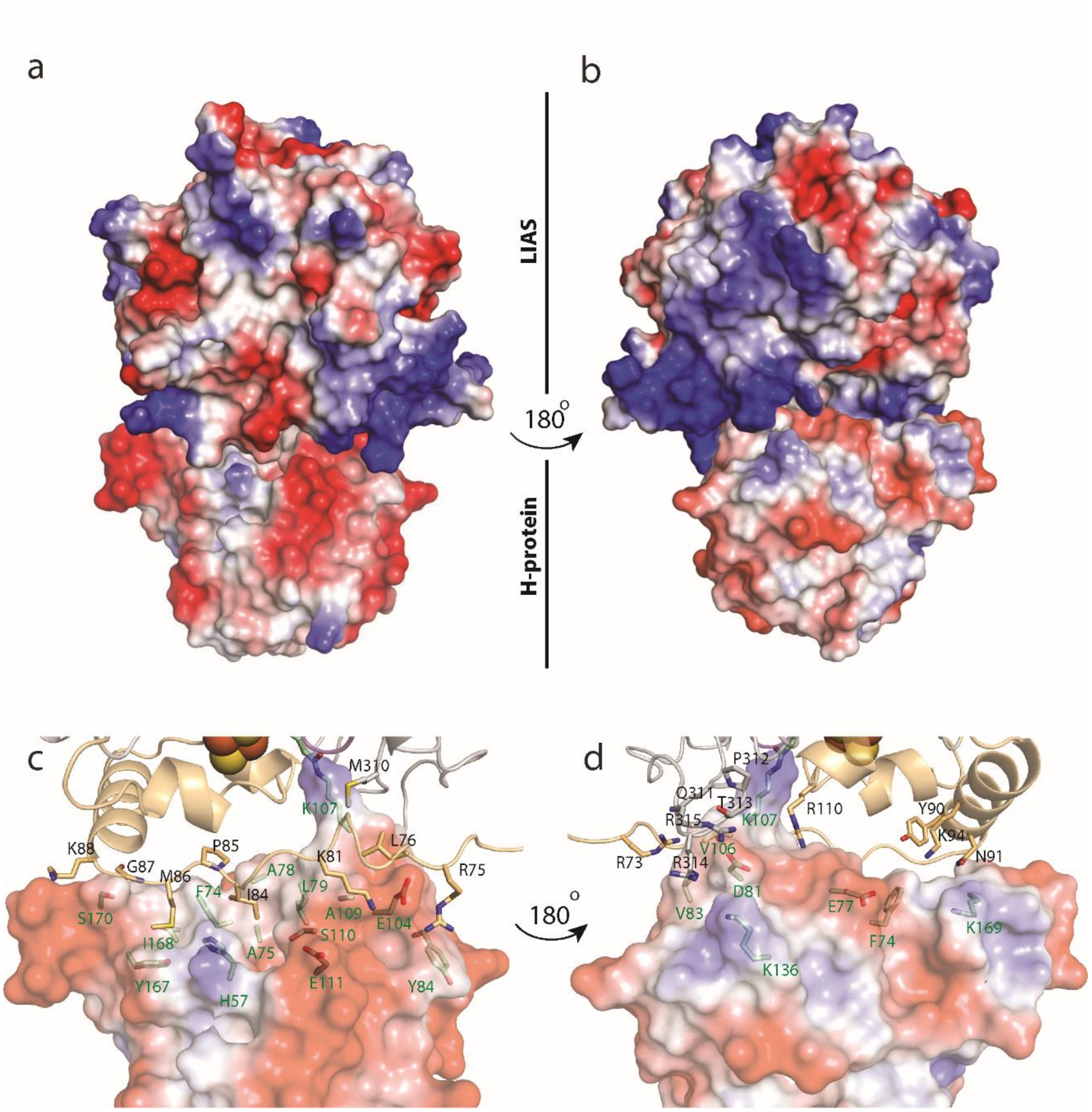

Supplementary Fig. 5. **Protein-protein interactions between LIAS and H protein (H_{pro}) in the LIAS and 6-mercaptooctanoyllysyl (6S-OCT)-H_{pro} covalent complex.** Electrostatic surface potential of the complex (blue is positively charged while red is negatively charged) (**a**), and after a 180˚ rotation (**b**). LIAS residues involved in complex formation are shown as sticks (black numbering), color code: N-terminal domain is tan, radical SAM domain is light grey, and C-terminal domain is purple. Electrostatic surface potential with H-protein residues involved in complex formation represented by sticks (green numbering) (**c**), after 180˚ rotation (**d**). Detailed interactions between LIAS and the H_{pro} can be found in **Supplementary Table 2**.

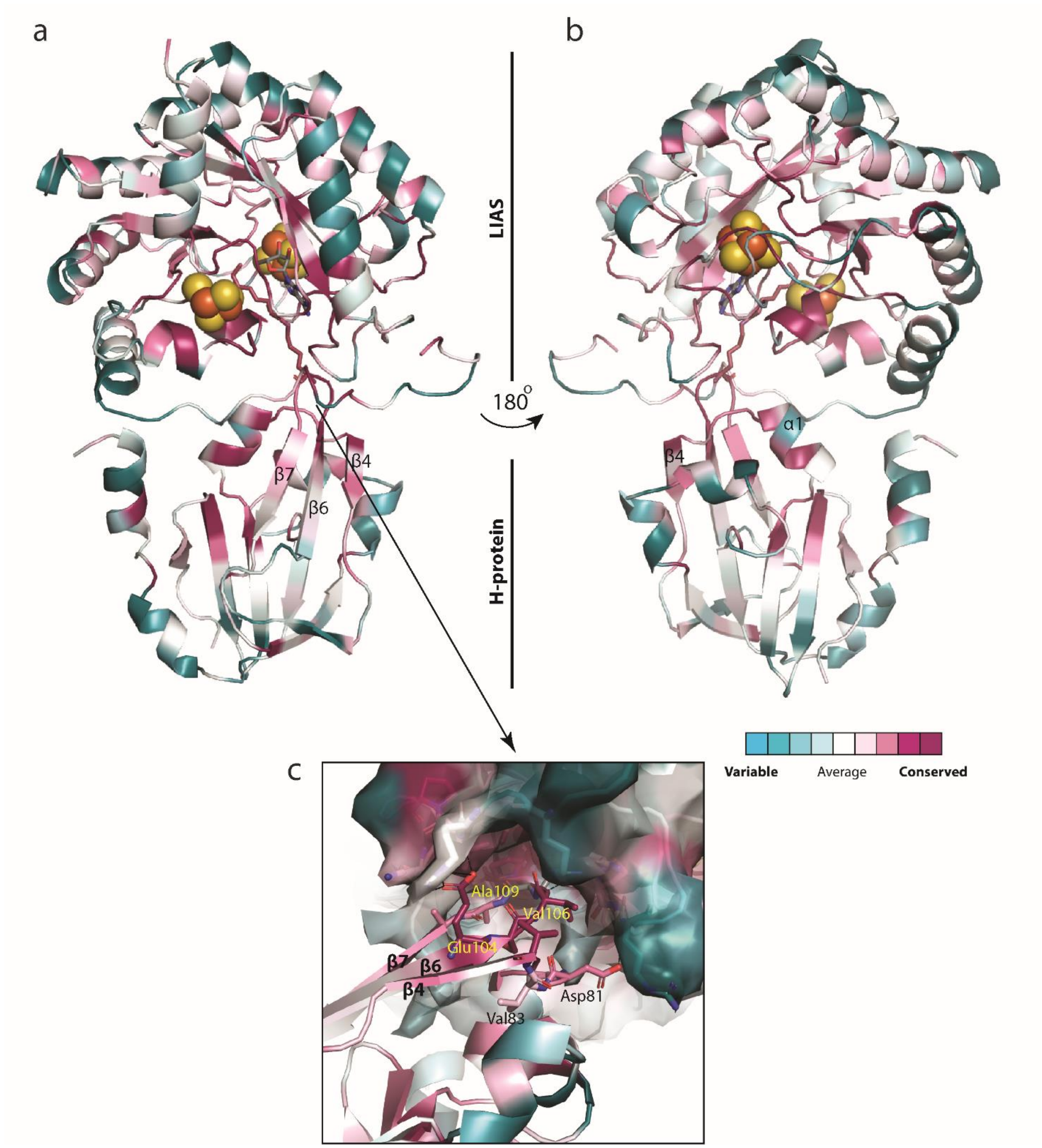

Supplementary Fig. 6. **Analysis of conserved residues in the LIAS and 6-mercaptooctanoyllysyl H-protein (6S-OCT-H_{pro}) covalent complex.** Cartoon representation of the LIAS and H_{pro} complex in the presence of 5'-5'-deoxyadenosine (dAH)+Met highlighting conserved residues. The coloring corresponds to the conservation of the amino acids calculated with the Consurf server (*1*). Overall structure of the complex (**a**) and after a 180˚ rotation (**b**). A representation of H_{pro} residues (cartoon) involved in complex formation with LIAS (surface) (**c**).

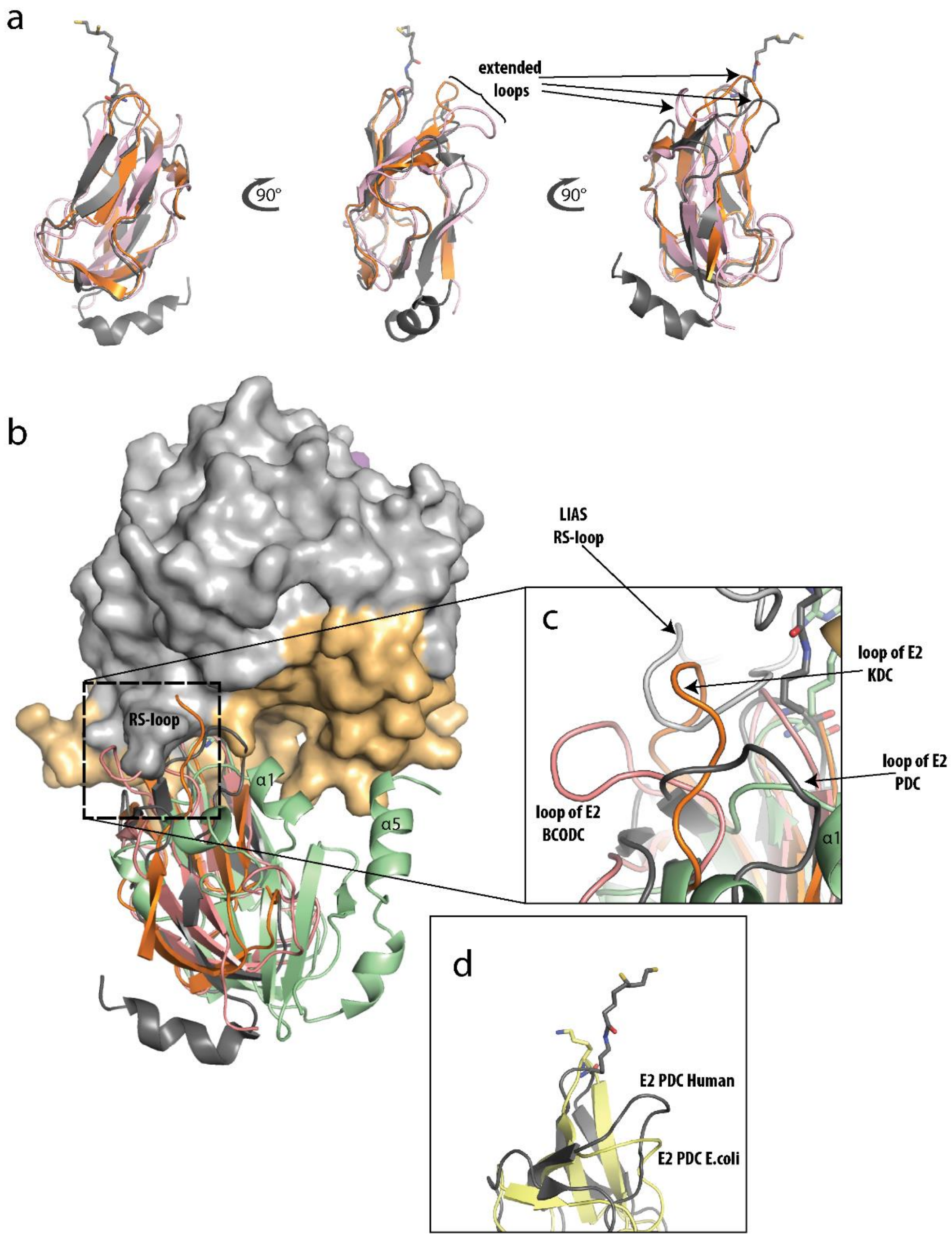

89 **Supplementary Fig. 7**. **Superimposed structures of the E2 subunits of the branch chain oxo-acid dehydrogenase complex (BCODC), α-ketoglutarate dehydrogenase complex (KDC), and pyruvate dehydrogenase complex (PDC).** A cartoon representation of the overlaid E2 subunits of the BCODC, light pink color, PDB ID: 1K8M; KDC, orange color (AlphaFold prediction); PDC, dark gray color, PDB ID: 1Y8O (**a**). The superimposed E2 subunits and the H-protein (H_{pro}) (light green) in complex with LIAS are shown as a surface: N-terminal domain (tan), radical SAM (RS) domain (grey), and C-terminal helix (purple) (**b**). The clash region between LIAS and the E2 subunits of the BCODC, KDC, and PDC (**c**). The superimposed structures of the E2 subunits from the human PDC (dark gray; PDB ID: 1Y8O) and *E. coli* PDC (yellow; PDB ID: 1QJO) (**d**).

90

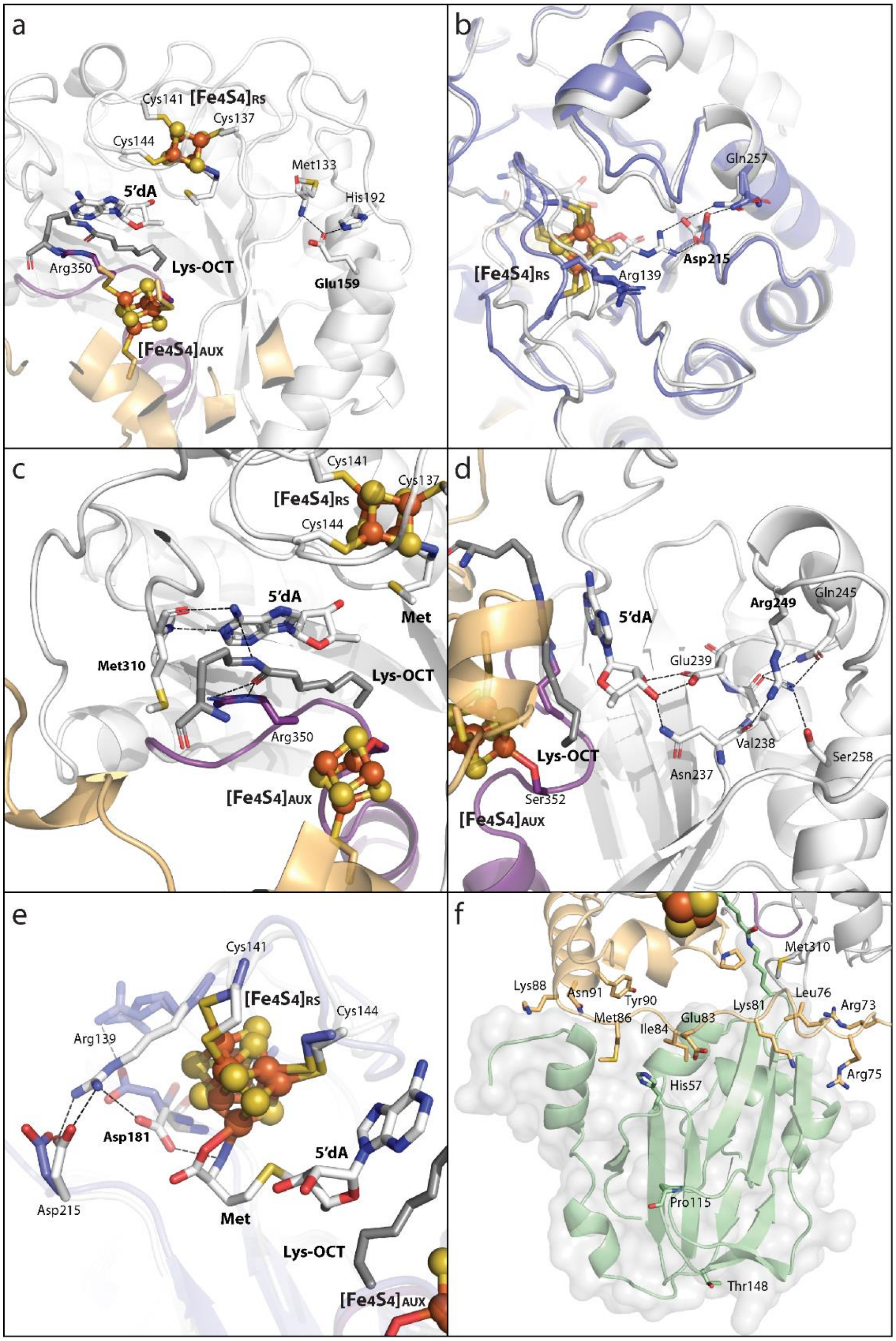

Supplementary Fig. 8. **The interaction of conserved residues of LIAS involved in Nonketotic Hyperglycinemia (NKH)**. The interaction of the conserved residues of LIAS: Glu159 (**a**), Asp215 (**b**) Arg249 (**c**) and Met310 (**d**) whose mutations are associated with NKH. LIAS color code: N-terminal domain (tan); radical SAM domain (light grey); C-terminal helix (purple); iron–sulfur atoms of the auxiliary ($[Fe_4S_4]_{AUX}$) and radical SAM ($[Fe_4S_4]_{RS}$) clusters are represented as orange and yellow balls, respectively. A cartoon representation of the structure of LIAS in the presence of the octanoylated 8_{mer} peptide substrate (OCT-8_{mer}), 5'-deoxyadenosine (5'-dAH)+Met (**a, c, d, e**), and superimposed with the structure of LIAS in the presence dithiothreitol (DTT) (**b, e**) (blue color). The interaction of LIAS and octanoylated H-protein (OCT-H_{pro}) (green, **f**), demonstrating the positions of altered residues His57, Pro115 and Thr148 of H_{pro}.

Supplementary References

1. H. Ashkenazy *et al.*, ConSurf 2016: an improved methodology to estimate and visualize evolutionary conservation in macromolecules. *Nucleic Acids Res* **44**, W344-350 (2016).
2. N. D. Lanz *et al.*, RlmN and AtsB as models for the overproduction and characterization of radical SAM proteins. *Methods Enzymol.* **516**, 125-152 (2012).
3. M. R. Wilkins *et al.*, Protein identification and analysis tools in the ExPASy server. *Methods Mol Biol* **112**, 531-552 (1999).
4. M. Bradford, A rapid and sensitive method for the quantitation of microgram quantities of protein utilizing the principle of protein dye-binding. *Anal. Biochem.* **72**, 248-254 (1976).
5. N. D. Lanz *et al.*, Characterization of Lipoyl Synthase from *Mycobacterium tuberculosis*. *Biochemistry* **55**, 1372-1383 (2016).
6. R. M. Cicchillo *et al.*, Lipoyl synthase requires two equivalents of S-adenosyl-L-methionine to synthesize one equivalent of lipoic acid. *Biochemistry* **43**, 6378-6386 (2004).
7. Z. Otwinowski, W. Minor, Processing of x-ray diffraction data collected in oscillation mode. *Methods Enzymol.* **2765**, 307–326 (1997).
8. W. Minor, M. Cymborowski, Z. Otwinowski, M. Chruszcz, HKL-3000: the integration of data reduction and structure solution--from diffraction images to an initial model in minutes. *Acta crystallographica. Section D, Biological crystallography* **62**, 859-866 (2006).
9. A. G. Torres, E. Batlle, L. R. de Pouplana, Role of tRNA modifications in human diseases. *Trends Mol. Med.* **20**, 306–314 (2014).
10. T. C. Terwilliger *et al.*, Iterative model building, structure refinement and density modification with the PHENIX AutoBuild wizard. *Acta crystallographica. Section D, Biological crystallography* **64**, 61-69 (2008).
11. A. J. McCoy *et al.*, Phaser crystallographic software. *J. Appl. Crystallogr.* **40**, 658–674 (2007).
12. P. Emsley, K. Cowtan, Coot: model-building tools for molecular graphics. *Acta Crystallogr. D Biol. Crystallogr.* **60**, 2126–2132 (2004).
13. P. Emsley, B. Lohkamp, W. G. Scott, K. Cowtan, Features and development of Coot. *Acta Crystallogr. D Biol. Crystallogr.* **66**, 486–501 (2010).
14. P. V. Afonine *et al.*, Towards automated crystallographic structure refinement with phenix.refine. *Acta crystallographica. Section D, Biological crystallography* **68**, 352-367 (2012).
