# Supplementary material for: Structural basis for catalysis by human lipoyl synthase
Source: Nat Commun. 2025 Jul 10;16:6355. doi: 10.1038/s41467-025-61393-x (PMC12246082; doi:10.1038/s41467-025-61393-x)
Supplement: Supplementary file 2 — Reporting Summary [file 41467_2025_61393_MOESM2_ESM.pdf]

nature portfolio

Corresponding author(s): Squire J.Booker

Last updated by author(s): Apr 29, 2025

Reporting Summary

Nature Portfolio wishes to improve the reproducibility of the work that we publish. This form provides structure for consistency and transparency in reporting. For further information on Nature Portfolio policies, see our Editorial Policies and the Editorial Policy Checklist.

Statistics

For all statistical analyses, confirm that the following items are present in the figure legend, table legend, main text, or Methods section.

n/a	Confirmed	
☐	☒	The exact sample size (*n*) for each experimental group/condition, given as a discrete number and unit of measurement
☐	☒	A statement on whether measurements were taken from distinct samples or whether the same sample was measured repeatedly
☒	☐	The statistical test(s) used AND whether they are one- or two-sided<br>*Only common tests should be described solely by name; describe more complex techniques in the Methods section.*
☒	☐	A description of all covariates tested
☒	☐	A description of any assumptions or corrections, such as tests of normality and adjustment for multiple comparisons
☒	☐	A full description of the statistical parameters including central tendency (e.g. means) or other basic estimates (e.g. regression coefficient) AND variation (e.g. standard deviation) or associated estimates of uncertainty (e.g. confidence intervals)
☒	☐	For null hypothesis testing, the test statistic (e.g. *F*, *t*, *r*) with confidence intervals, effect sizes, degrees of freedom and *P* value noted<br>*Give P values as exact values whenever suitable.*
☒	☐	For Bayesian analysis, information on the choice of priors and Markov chain Monte Carlo settings
☒	☐	For hierarchical and complex designs, identification of the appropriate level for tests and full reporting of outcomes
☒	☐	Estimates of effect sizes (e.g. Cohen's *d*, Pearson's *r*), indicating how they were calculated

Our web collection on statistics for biologists contains articles on many of the points above.

Software and code

Policy information about availability of computer code

Data collection	HKL2000/3000 packages were used for data processing of LIAS and LIAS-Hprotein complex. SAD, Phaser 2.8.3 and Autobuild were used.
Data analysis	The Phenix sute v1.20.1-4487 (including Phenix.refine, Autosol, Autobuild, etc) was used for crystallography data refinement. Coot 0.9.8.1 was used for crystallography modeling. PyMOL 2.5.2 was used to make crystallography figures.

For manuscripts utilizing custom algorithms or software that are central to the research but not yet described in published literature, software must be made available to editors and reviewers. We strongly encourage code deposition in a community repository (e.g. GitHub). See the Nature Portfolio guidelines for submitting code & software for further information.

Data

Policy information about availability of data

All manuscripts must include a data availability statement. This statement should provide the following information, where applicable:

- Accession codes, unique identifiers, or web links for publicly available datasets
- A description of any restrictions on data availability
- For clinical datasets or third party data, please ensure that the statement adheres to our policy

Atomic coordinates and structure factors for the reported crystal structures in this work have been deposited to the Protein Data Bank (PDB) under the accession numbers 8TRW (LIAS); 8TSK (LIAS + N-octanoylllysyl-8mer peptide + 5'-dAH); 8UGO (LIAS-6S-OCT-Hprotein complex + 5'-dAH+ Met) and 9C19 (LIAS-6S-OCT-Hprotein complex + SAM).

Research involving human participants, their data, or biological material

Policy information about studies with human participants or human data. See also policy information about sex, gender (identity/presentation), and sexual orientation and race, ethnicity and racism.

Reporting on sex and gender	Reporting on sex and gender is not applicable to this study because no human participants were involved.
Reporting on race, ethnicity, or other socially relevant groupings	Reporting on race, ethnicity, or other socially relevant groupings is not applicable to this study because no human participants were involved.
Population characteristics	Reporting on race, ethnicity, or other socially relevant groupings is not applicable to this study because no human participants were involved.
Recruitment	No human participants were recruited for this study
Ethics oversight	No ethics oversight was included in this study because no human participants were involved.

Note that full information on the approval of the study protocol must also be provided in the manuscript.

Field-specific reporting

Please select the one below that is the best fit for your research. If you are not sure, read the appropriate sections before making your selection.

[x] Life sciences [ ] Behavioural & social sciences [ ] Ecological, evolutionary & environmental sciences

For a reference copy of the document with all sections, see nature.com/documents/nr-reporting-summary-flat.pdf

Life sciences study design

All studies must disclose on these points even when the disclosure is negative.

Sample size	Structures from individual protein crystals were initially solved by SAD phasing or by molecular replacement for subsequent crystals. Sample size calculation is not applicable to this study, because single protein crystals were solved.
Data exclusions	Anomalous and native LIAS datasets were integrated and scaled using the HKL2000 or HKL3000 package. No data exclusions were applied.
Replication	Replication is not applicable to this study, because single protein crystals structures were solved.
Randomization	Randomization is not applicable to this study, because single protein crystal structures were solved.
Blinding	No crystal structure of this enzyme exists and as such, no expectations were made on how it would appear. Thus, blinding was not necessary for this study.

Reporting for specific materials, systems and methods

We require information from authors about some types of materials, experimental systems and methods used in many studies. Here, indicate whether each material, system or method listed is relevant to your study. If you are not sure if a list item applies to your research, read the appropriate section before selecting a response.

Materials & experimental systems

n/a	Involved in the study
[x]	[ ] Antibodies
[x]	[ ] Eukaryotic cell lines
[x]	[ ] Palaeontology and archaeology
[x]	[ ] Animals and other organisms
[x]	[ ] Clinical data
[x]	[ ] Dual use research of concern
[x]	[ ] Plants

Methods

n/a	Involved in the study
[x]	[ ] ChIP-seq
[x]	[ ] Flow cytometry
[x]	[ ] MRI-based neuroimaging

Plants

Seed stocks	No seeds were used in this study.
Novel plant genotypes	No plants were used in this study.
Authentication	No plants or seeds were used in this study.
